# Acceptability of an mHealth App for Monitoring Perinatal and Postpartum Mental Health: Qualitative Study With Women and Providers

**DOI:** 10.2196/44500

**Published:** 2023-06-07

**Authors:** Deepthi S Varma, Maya Mualem, Amie Goodin, Kelly K Gurka, Tony Soo-Tung Wen, Matthew J Gurka, Kay Roussos-Ross

**Affiliations:** 1 Department of Epidemiology College of Public Health and Health Professions, College of Medicine University of Florida Gainesville, FL United States; 2 Pharmaceutical Outcomes and Policy, Center for Drug Evaluation and Safety Consortium for Medical Marijuana Clinical Outcomes Research College of Pharmacy, University of Florida Gainesville, FL United States; 3 Division of Maternal and Fetal Medicine, Department of Obstetrics and Gynecology College of Medicine, University of Florida Gainesville, FL United States; 4 Department of Pediatrics College of Medicine, University of Florida Gainesville, FL United States; 5 Department of Obstetrics and Gynecology College of Medicine, University of Florida Gainesville, FL United States; 6 Department of Psychiatry College of Medicine, University of Florida Gainesville, FL United States

**Keywords:** perinatal mental health, mobile health, mHealth, mobile apps, ecological momentary assessment, EMA, mobile phone

## Abstract

**Background:**

Up to 15% of pregnant and postpartum women commonly experience undiagnosed and untreated mental health conditions, such as depression and anxiety, which may result in serious health complications. Mobile health (mHealth) apps related to mental health have been previously used for early diagnosis and intervention but not among pregnant and postpartum women.

**Objective:**

This study aims to assess the acceptability of using mHealth to monitor and assess perinatal and postpartum depression and anxiety.

**Methods:**

Focus group discussions with pregnant and postpartum women (n=20) and individual interviews with health care providers (n=8) were conducted to inform the acceptability of mHealth and determine its utility for assessing perinatal and postpartum mood symptoms. Participants were recruited via purposive sampling from obstetric clinics and the surrounding community. A semistructured interview guide was developed by an epidemiologist with qualitative research training in consultation with an obstetrician. The first author conducted all focus group discussions and provider interviews either in person or via Zoom (Zoom Video Communications, Inc) depending on the COVID-19 protocol that was in place during the study period. All interviews were audio recorded with consent; transcribed; and uploaded for coding to ATLAS.ti 8 (ATLAS.ti Scientific Software Development Gmb H), a qualitative data analysis and retrieval software. Data were analyzed using the deductive content analysis method using a set of a priori codes developed based on the interview guide. Methodological rigor and quality were ensured by adopting a systematic approach during the implementation, data collection, data analysis, and reporting of the data.

**Results:**

Almost all women and providers had downloaded and used at least 1 health app. The respondents suggested offering short questions in layperson language that could be understood by women of all educational levels and offering no more than 2 to 3 assessments per day at preferred timings decided by the women themselves. They also suggested that the women themselves receive the alerts first, with other options being family members, spouses, or friends if the women themselves did not respond within 24 to 72 hours. Customization and snooze features were strongly endorsed by women and providers to improve acceptability and utility. Women mentioned competing demands on their time during the postpartum period, fatigue, privacy, and the security of mental health data as concerns. Health care professionals highlighted the long-term sustainability of app-based mood assessment and monitoring as an important challenge.

**Conclusions:**

The findings from this study show that mHealth would be acceptable to pregnant and postpartum women for monitoring mood symptoms. This could inform the development of clinically meaningful and inexpensive tools for facilitating the continuous monitoring of, the early diagnosis of, and an early intervention for mood disorders in this vulnerable population.

## Introduction

### Background

Perinatal depression and anxiety substantially affect the health of women and their offspring. Approximately 10% to 15% of adult mothers are diagnosed with postpartum depression [1]. In a recent study in the United States, the overall prevalence of postpartum depression was 13%, ranging from 10% in Illinois to 24% in Mississippi [2]. Many women taking psychiatric medications discontinue treatment during pregnancy owing to fears of possible danger to their fetuses [3]. However, women who discontinue these medications during pregnancy are 5 times more likely to experience a relapse of depression during pregnancy or the postpartum period [4]. Perinatal depression is a leading cause of maternal morbidity and mortality, and untreated depressive symptoms can persist for 3 years after giving birth [5-7]. If left untreated, postpartum depression can lead to poor medication adherence, poor nutrition, self-harm, suicide, homicide, overdose, and an exacerbation of preexisting chronic medical conditions [5,6]. Women with mild symptoms of depression or anxiety during pregnancy or post partum may fail to recognize these symptoms or choose not to disclose them because of stigma, shame, or the fear of losing custody of their newborn [8].

The American College of Obstetrics and Gynecology recommends the consideration of the “4th trimester” (ie, from delivery through 3 months post partum) as a critical time for the continued engagement of the postpartum woman. Recommendations for obstetrician-gynecologists (OBGYNs) and other obstetric care providers are to screen patients for mood disorders at least once during the perinatal period and again during the postpartum visit using validated tools [9]. The current model of a single postpartum follow-up visit for postpartum care (covered by insurance) is inadequate to monitor changes in mental health. Maternal mortality or morbidity is exacerbated by the woman’s inability to recognize early warning signs during the postpartum period and seek immediate treatment either because of her lack of education regarding the symptoms or the stress and lifestyle changes associated with being a new mother. We believe that a mobile health (mHealth) app that uses ecological momentary assessment (EMA) technology can effectively help diagnose, monitor, and assess postpartum mood symptoms in women during the time between their discharge and postpartum visit.

### Benefits of EMA Technology

EMA is an efficient method for generating data on people’s positive and negative affect, self-harm behavior, and other symptoms associated with mood disorders in real time in their natural environment [10,11]. EMA technology may reduce the impact of bias on self-report and allows the examination of within-participant processes through repeated assessments. Furthermore, the real-time reporting of symptoms, effects, and behaviors complements existing assessment methods by providing a trajectory of mood symptoms between 2 clinical appointments [12]. When applied effectively, EMA technology can assist with early symptom recognition, facilitate treatment, and potentially reduce severe maternal morbidity and mortality. Recent studies on mood assessment using smartphone apps with EMA technology have reported substantial compliance with the smartphone apps [13-15]. In addition, EMA technology also assists in improving the awareness of patients about their mood fluctuations and their context, which, in turn, can help them manage their symptoms more efficiently and seek care [16].

### Goal of This Study

However, only a few studies have used EMA technology to monitor and assess perinatal and postpartum mood disorders. We propose to develop and pilot-test a smartphone-based app that uses EMA technology to assess mood symptoms among postpartum women for a period of 6 weeks. To facilitate the successful implementation of such a pilot intervention, it is important to understand the acceptability of such a technology among providers and patients. Therefore, the objective of this study was to characterize the acceptability of using an EMA technology–enabled mHealth app to monitor mood among pregnant and postpartum women and to explore the acceptability of its use.

## Methods

### Overview

A total of 7 focus group discussions (FGDs) were conducted among 20 pregnant or postpartum women, and 8 one-on-one interviews were conducted with health care providers. Purposive sampling was used to recruit ethnically diverse participants of different ages from varied socioeconomic backgrounds to facilitate the development of culturally sensitive content for the app. This study was approved by the University of Florida institutional review board (IRB).

### Recruitment

#### Women

Pregnant and postpartum (within 1 day to 1 year after giving birth) women were recruited from the community through flyers posted in local obstetrics clinics and community women’s health clinics and Healthy Start, a care coordination program for families with young children at risk for poor health and developmental outcomes. Participants were also recruited through local community engagement programs. Eligibility criteria included age of ≥18 years, current or recent pregnancy (having a child aged ≤3 years), ability to provide consent to participate, and ability to read and write in English. Interested women who contacted the study coordinator by calling the number on the flyer were screened for eligibility. The coordinator provided eligible women with additional information about the study and several options for the day and time of the FGD. The participants were sent a reminder of the selected time and location of their FGD.

#### Providers

Providers were recruited from an obstetric practice in a university academic setting and a private practice clinic that focuses on treatment for maternal mental health. Interviews were advertised via flyers distributed to the staff by office managers. The eligibility criteria included providing obstetric or psychiatric care to pregnant or postpartum women and the ability to read and write in English. Interested providers also contacted the study coordinator to receive additional information about the study and schedule the interview. The participants were then sent a Zoom (Zoom Video Communications, Inc) link with a password for the interview.

### Instruments

The FGD and semistructured interview guides were developed after an extensive literature review and discussion with experts in the fields of mobile phone–based apps and perinatal mental health, including mental health professionals and OBGYNs. The topics included previous experience with mHealth apps, the types of questions asked through the app, the acceptable frequency of alerts, the predictions of patterns of use of the app, notification logistics, benefits, and challenges.

Before starting the discussion, both sets of respondents were given a detailed explanation of an mHealth app that was being planned for development and the various features that could be incorporated into the app. The respondents were requested to reflect on the various proposed features, inform the interviewers whether the features were acceptable, and provide the interviewers with suggestions for improvement. Textbox 1 provides a list of the topics included in the interview guide.

**List of topics included in the interview guide.**

Mobile phone–based apps that monitor health or manage symptoms

- General opinion on mobile health apps
- User experience with such apps

Usability of a mobile phone–based app for monitoring specifically mood changes and anxiety symptoms among pregnant and postpartum women

- Do you consider mobile phone–based apps as a good method for assessing women’s anxiety and mood symptoms during pregnancy? Please explain your response with reasons.
- What are your opinions regarding the benefits of such an app for women in terms of self-monitoring symptoms?
- What are your opinions regarding the benefits of such an app for women in terms of accessing early interventions?

Propose app description: Briefly describe the proposed mobile phone app and its functions and then ask the following questions:

- What are your opinions regarding the appropriateness of these questions (in terms of language, relevance, and the ability to assess symptoms)?
- What are your opinions regarding repeated assessments via SMS text messages throughout the day?
- How many times in a day can the app send questions?
- What is the best time gap between questions throughout the day?
- What are the best times of the day to get a maximum response?
- Perceived usefulness of such assessments for helping women feel supported by their health care team
- Possibility of missing such repeated questions throughout the day for a prolonged period of 6 to 8 months during their pregnancy and post partum
- Acceptance of such repeated assessments by their family members

Alerts that will be generated by the app in response to your answers to the questions asked

- When could the app start sending alerts to providers?
- Which are the types of questions and responses that should trigger an alert?
- Do women also require alerts or should alerts be sent only to providers?
- What are the frequency and duration of alerts sent to women?
- What do you think is the best first response from your provider after receiving an alert about your mood change?

### Data Collection

Pregnant and postpartum participants received 2 reminders of their scheduled FGDs, a week before and the day before the discussion. FGDs were restricted to a maximum of 6 participants each. All in-person FGDs were conducted in a private room behind closed doors at HealthStreet, the community engagement facility of University of Florida. Upon arrival at the site, the participants provided consent, were assigned a unique ID for addressing them during the FGD to ensure anonymity, and provided a sociodemographic questionnaire to complete before starting the FGD. A discussion leader conducted the FGDs using the interview guide in a private room, free of outside distraction, and each FGD was audio recorded. In addition to the topics included in the guide, unanticipated areas of interest were discussed as they arose. Each FGD was approximately 60 minutes in duration.

The providers received a reminder the day before the interview. Owing to COVID-19–related restrictions implemented by the IRB, all 8 provider interviews were conducted via Zoom after the administration of the sociodemographic questionnaire. Each semistructured interview was recorded and approximately 30 minutes in duration.

The FGDs and in-depth interviews were conducted by the first author, who is a qualitative researcher with extensive experience in successfully implementing qualitative research protocols and has published several peer-reviewed qualitative research studies [17,18].

### Data Analysis

All the recordings were transcribed and uploaded to ATLAS.ti (ATLAS.ti Scientific Software Development Gmb H), a qualitative data analysis and retrieval software, for deductive content analysis [19]. An a priori list of codes was developed based on the interview guide. The first and second authors identified and added new codes via open coding during the analysis. Both coders met several times during the coding process to discuss the appropriateness and definitions of the new codes. Recurring patterns and themes were identified by examining and categorizing the codes generated in the first coding cycle. Methodological rigor and quality were ensured by adopting a systematic approach during the implementation, data collection, data analysis, and reporting of the data.

### Ethics Approval, Informed Consent, and Participation

The University of Florida Institutional Review Board approved this study (Study ID: IRB201801910). A University of Florida IRB–approved signed informed consent form was obtained from all the participants before the data collection. All the audio recordings were assigned a unique ID, which was known only to the principal investigator (first author) and research coordinator (second author). All participants received a US $20 Amazon gift card for their time and participation.

## Results

### Sociodemographic Characteristics

#### Women

The mean age of the women participants was 31.6 (SD 5.05) years. Of the 20 women participants, 11 (55%) identified as White, 4 (20%) identified as Black or African American, 1 (5%) as “other race,” 1 (5%) as Hispanic or Latina, and 4 (20%) did not report their race or ethnicity. Most of the women participants (13/20, 65%) had ≥12 years of education. Overall, 65% (13/20) of women were married, and 50% (10/20) of women had either full- or part-time employment. Of the 20 women, 5 (25%) reported having ≥2 children, whereas the remainder (15/20, 75%) reported having only 1 child at the time of the study.

#### Providers

All 8 providers were women and had ≥18 years (master) of education. Among the 8 providers, 6 (75%) identified as White, 1 (12%) identified as Black or African American, and 1 (12%) identified as Hispanic or Latina. Of the 8 providers, 1 (12%) was an OBGYN, 2 (25%) were nurse practitioners, 2 (25%) were mental health therapists, and 3 (38%) were clinical social workers. The therapists and clinical social workers specialized in the area of perinatal and postpartum mental health. The OBGYN and the 2 nurse practitioners worked in a women’s clinic, which also offered mental health services to pregnant and postpartum women. The clinical social workers were employed at a community-based mental health clinic.

Most analysis codes were common between both interview groups, FGDs and provider interviews, and some codes were specific to the individual interview data. Table 1 provides a list of the themes, codes, and definitions used in this study.

**Table 1:** List of themes, codes, and definitions used in this manuscript.

Themes and codes			Definitions
**Experience with health apps**			
	Health app experience	Experience and duration of using health apps in the past year	
	Opinions on health apps	Opinions on any type of health apps on smartphones	
**mHealth^a^ app features **			
	Question format	The format or style in which the questions are asked	
	Question appropriateness	The appropriateness of the questions in terms of language, relevance, and the ability to assess symptoms	
	Question frequency	The number of times the woman would like to receive questions from the app	
	Alert trigger	The types of responses to questions that should trigger an alert	
	Alert recipient	Whether the woman should also receive the alert or the alert should be sent only to the provider and which provider the woman wants to receive the alerts	
	Alert frequency	The frequency and duration of alerts sent to the woman	
	Question time frame	The amount of time women would like between questions throughout the day	
	Question timing	The best time of day to ask the questions when women are most likely to respond	
**Notification logistics**			
	Customization	Being able to personalize features in the app to fit individual needs	
	Snooze function	A function of the app that allows users to delay a question to answer it when they are free	
**Acceptability of mHealth apps**			
	Benefits: utility in assessing mood	Whether the woman views phone-based apps as a good way to assess women’s anxiety and mood symptoms during pregnancy	
	Benefits: utility in self-monitoring symptoms	The benefits of using an app for women to self-monitor symptoms of mood and anxiety	
	Benefits: utility in early intervention	The benefits of using an app for women to access early intervention of health care services	
	Benefits: usefulness	Perceived usefulness of assessments for making women feel supported by their health care team	
	Benefits: maternal mental health stigma	Whether the woman believes there is a stigma associated with maternal mood and anxiety during and after pregnancy	
	Challenges: family acceptance	The likelihood of family members’ acceptance of women having to answer repeated mood assessments throughout the day	
	Challenges: utility for substance-using pregnant women	The likelihood of substance-using pregnant women using an app such as this	
	Challenges: missing questions	The likelihood of missing repeated questions throughout the day for a prolonged period of 6 to 8 months during pregnancy and post partum	

^a^mHealth: mobile health.

### Experience With Mobile Phone–Based Health Apps

Almost all women and providers had downloaded and used at least 1 health app on their mobile phones and found it useful. They all agreed that mHealth apps are an emerging way to monitor health:

I actually just got an app “Ovia pregnancy.” Every two/three days they update you with new information as well as every single day they pop up on your phone and it says some questions [such as] “did you take your prenatal today?” and you could just click yes or no...

Woman, focus group (FG) 1

I have an app for developmental for children so that helps a lot...then I had the “Ovia Pregnancy” and I also had a “Seizure Tracker” due to the fact I have seizures so that helps with tracking the seizure.

Woman, FG 1

I had “Myfitness Pal” and a food app “Nutrition Plus” where I was trying to log food. I also used the “Beach Body” app for like two months and then “Myfitness Pal” maybe a week. [laughs] Not very long.

Provider, registered nurse, OBGYN clinic

### mHealth App Features

After briefly explaining the various features of a mHealth appl that uses EMA technology, all women and providers were asked a series of questions regarding the various features that they would like to incorporate into the mHealth app.

#### Type and Language of Assessment Questions

Women and clinicians reported that the optimal formatting for a mobile app assessment is short questions in layperson’s language, which women of all educational levels could understand and respond to appropriately:

I would just keep it [the questions] very simple...and short.

Provider, OBGYN

If it is just like one simple question, indicate on a scale of one to five, or how are you feeling right now? I think women are more likely to do something like that.

Woman, FG 8

I prefer to just be able to click yes/no or swipe or [check] a smiley face, sad face...or something quick like that...

Woman, FG 5

A woman mentioned that it would be helpful and encouraging if the app could occasionally send pictures to cheer up the user:

Even random encouraging little things...not always are you sad? Just kind of send a pop-up picture like...“hey, look at this cute little cat and be happy right now”...

Woman, FG 5

#### Frequency and Timing of Questions

Several women and providers mentioned morning and evening as the best times to receive responses:

...morning and night, you know. “How do you feel when you wake up?” Are you in a good mood when you wake up or are you in a bad mood? Before you go to bed, “How was your day?”...I feel like that might be better.

Woman, FG 2

I would say probably morning and then probably bedtime.

Provider, OBGYN

Women and providers stated that they would be willing to answer 2 to 3 questions per day:

Personally, I think that I would ignore many of the questions if there were like five questions a day.

Woman, FG 2

I would say no more than two to three questions.

Provider, licensed social worker, community-based mental health clinic

I would think about three times maybe every three to four hours.

Woman, FG 1

#### Alert Trigger

The participants were presented with a feature of the app for triggering an alert to a person of the woman’s choice if the woman did not respond to the questions sent through the app. All the participants mentioned that the woman herself should receive the first alert, giving her enough time to act based on the alert. Family members, spouses, or friends were additional choices for alert recipients if the woman did not respond. Several women reported that they were not comfortable with the app sending an alert directly to the provider if they did not respond:

I think realistically for someone like a family member or husband [should get the alert] in 24 hours...giving just a little reminder for them to remind the woman...and then to the physician for professional help in about 72 hours.

Woman, FG 11

I think first person for me would be spouse and then second would be therapist.

Woman, FG 9

If I had to assign someone, I would rather assign a person who could check on me soon...before...[going to the provider].

Woman, FG 5

I think three [unanswered questions]...maybe to the provider first, so that the provider can speak with the client and then involve the family if needed.

Provider, LSW, community-based mental health clinic

I think it depends on the person. They could have an option for a family member or partner and a provider...but if they had the option, I think all three options [the woman herself, a family member, or a provider] would be great and then they could just pick what one would be alerted.

Provider, LSW, c-based mental health clinic

Although most providers thought that receiving alerts would be beneficial, many expressed a concern that health care professionals would be unable to respond immediately because of competing demands:

The attending [nurse] will get a message and then they will contact us. But sometimes when you have so many providers and patients and such a big system, a lot of things get lost.

Provider, nurse practitioner, OBGYN clinic

If she sends an alert in the middle of the night and you’re not on the computer, you don’t open the EPIC (Electronic Health Record software) until the next day...that will be a problem.

Provider, nurse practitioner, OBGYN clinic

Approximately 24 to 72 hours was mentioned as the ideal wait time for unanswered questions to trigger an alert. Most women stated that they could have a busy day and might not have time to respond to the assessment questions; therefore, they would like the app to wait a day or 2 before triggering an alert.

#### Notification Logistics

The main function that was explored under notification logistics was the “snooze” function to identify how many times and how frequently women would like to receive reminders regarding assessment questions. All the participants agreed on the need for a “snooze” function but had mixed responses regarding the duration between the snoozes. Some women stated that they would like 30 minutes before the question reappears, whereas few others stated they would prefer the question to reappear after an hour or 2. Most women and providers suggested that the question reappear only once, if not answered the first time:

If it was thirty minutes, I don’t think that’s enough time to finish breastfeeding or changing a diaper or putting the child down...sometimes I think an hour or maybe two hours would be good.

Woman, FG 9

I feel like it [should] be at least five minutes to pass before it [the question notification] comes back again.

Provider, nurse practitioner, OBGYN clinic

In addition, most of the women and providers mentioned that they would like to have a “customization feature” that would help them set the snooze function as per their changing schedules:

I would like that if I had the choice in an application where I can choose to increase or decrease frequencies [of notifications]...if I could choose to increase when I am not feeling myself...maybe I want ten times a day.

Woman, FG 5

Actually the app should allow to put in the hours [when] it would be good for you to be reminded or approximate time period of the day...maybe customize each person’s needs.

Woman, FG 1

If they know that, they [woman] do not wake up until 10 o’clock and hence do not want her phone going off before 10 o’clock, being able to set up that preference in the app might be beneficial.

Provider, physician assistant, OBGYN clinic

### Acceptability of the Proposed mHealth App

#### Benefits

Women and providers mentioned that most family members would be accepting of women using a mobile phone app that assessed their mood and anxiety. Most women stated that their family members would be in support of such an app so that their mental health can be constantly monitored to ensure optimal health:

...it [mHealth app] would not bother him [her husband] and he would actually encourage it...

Woman, FG 3

I don’t know that you’d get too much pushback from spouses, particularly if the patient is already in mental health services. The family has some amount of acceptance and understanding of what’s going on.

Provider, LSW, community-based mental health clinic

Several women and providers mentioned that an app is beneficial for women to self-monitor their mental health and seek early diagnosis and intervention. Others stated that having the prompt care of a provider in terms of receiving immediate answers to their questions can be beneficial in reducing stress and would serve as a link between women and providers:

Moreover, that is really good sometimes to have that urgent right-off-the-bat help...or you don’t know what’s going to happen. You might get worse off and it is hard to juggle your mental health with children. So if you have somebody to reach out when you need it, that’s beneficial.

Woman, FG 1

I think if people are using the app they’re probably going to be more mindful and tuned in to their own self and what’s going on.

Provider, LSW, community-based mental health clinic

Another benefit mentioned is that the app will allow providers to save time and receive accurate information on their patients because the patients are logging their moods repeatedly, as opposed to being assessed just once when they go to the provider’s office:

That [app] would be so convenient. I think that would both save us time...[and] I would just have a more accurate assessment...

Provider, LSW, community-based mental health clinic

Another provider mentioned that the app will make women feel that they are in contact with the providers more than usual:

Anytime, in general, it is hard to spend a lot of time with your physician because physicians are busy, clinics are busy...this app would give them an additional interaction or whatever...where patients feel like they are cared about. Then if the messages also essentially goes to the provider that they may be more in contact with their provider then they would have otherwise been.

Provider, OBGYN

One of the providers mentioned that the potential benefits of using the mHealth app might serve as a motivation for the continuation of its use:

If patients feel, they are getting a benefit from doing it they would be likely to continue. If they do not feel they are getting a personal benefit that might be an issue for them.

Provider, OBGYN

#### Challenges

Few women stated that either they themselves or their family members might have concerns about entering personal health information into a mobile phone app owing to the fear of security breaches:

My husband would be concerned about privacy and on what was being done with the [personal] information...he hates Facebook...and he told me recently there was a [menstrual] period tracking app that was selling data to Facebook...

Woman, FG 1

My friends and family would say that is too invasive...[they say] “leave me alone I got it” or “I don’t need help.”

Woman, FG 5

I feel about 50% are gonna be understanding of it [the app] and then the other camp is gonna be like “Why do you need that...?” or “Why am I always getting these notifications?”...there’s going to be that half that’s just not [comfortable].

Provider, LSW, community-based mental health clinic

All the participants expressed concerns about pregnant and postpartum women who use substances not answering questions about substance use honestly owing to worries regarding the confidentiality of their responses and the fear of a Department of Children and Families investigation due to substance use:

I think substance abuse issues and being able to reliably log into something and do it again and again...I don’t know how the follow-through would happen

Woman, FG 11

I think that they would potentially have some concerns about privacy. If there’s someone who has an active legal case, perhaps that would be the person who would not be as willing.

Provider, LSW, community-based mental health clinic

Perinatal and postpartum women have busy schedules, including work, and sometimes additional children to take care of. Therefore, answering several questions through the app daily for 6 to 8 months was mentioned as a burden by a few:

Work hours is usually about 8 hours a shift so if I get called from these people [alert response via app] at work...I may possibly lose my job. They may ask what’s going on with my personal life or complain that too much of my personal life at job.

Woman, FG 1

I think there will be consistently missing data because of the nature of being pregnant and postpartum is so busy and so stressful...

Provider, LSW, community-based mental health clinic

In addition, one of the women expressed concern about keeping up with the iPhone (Apple Inc) or Android technology updates to ensure that the app runs smoothly:

Most apps are really useful, if they are able to keep up with the updates and technology because it [cell phones] are always sending updates...

Woman, FG 1

## Discussion

### Principal Findings

The findings from this study demonstrate that mHealth apps that use EMA technology are beneficial and acceptable to pregnant and postpartum women and their providers for monitoring mood and anxiety. Most women participants in this study had downloaded and used health apps during their prepregnancy, pregnancy, and postpartum periods, highlighting the fact that it is commonplace for all people to use smartphone-based apps to monitor and manage health [14]. Similar to earlier research findings, all women and providers acknowledged how challenging the postpartum period can be for women [20-22]. Ease or convenience in using an mHealth app was found to be an important theme throughout the discussion with women and providers. Regarding the daily assessments of mood through the mHealth app, both women and providers indicated their preference for simple questions in layperson language and the use of emojis such as smileys to report their daily mood and anxiety.

Furthermore, they also reported that a “menu of functions” from which they could select their choices is an important feature to improve the acceptability of the smartphone app. The “customization” of the app to meet the individual needs of end users was reported as a necessity for greater acceptability and sustained use by all women and providers. Previous studies have also reported that the “personalization” of health apps makes them more attractive to users [23,24]. Women cited features such as *snoozing* daily assessment questions, reminders, and alerts that must be customizable according to their changing needs during the postpartum period. Women preferred that alerts be sent to them, their spouse, or a trusted family member or friend, rather than to a provider, perhaps owing to the fear of stigmatization or mandatory reporting by providers, as has been reported in previous studies [25-27].

As in previous research, both women and providers in this study reported that the ability to self-monitor symptoms and take prompt action to prevent the worsening of their conditions through the use of mHealth is a benefit [28,29]. Mood is not a static phenomenon; it changes during the course of the day. Several women and providers highlighted the repeated real-time assessments using an app instead of in-person assessments during 1 or 2 postpartum visits as an important benefit of the app. Providers usually depend on patients’ self-report of symptoms over several weeks before the postpartum appointment. These reports are always subject to recall errors, especially those of postpartum women, who often experience fatigue associated with the care of a newborn. Therefore, providers were particularly interested in the availability of real-time data on the mood of their patients, which would provide more detailed and accurate information for diagnosis [30-32]. Real-time data not only may assist providers in the timely identification of concerning symptoms, as well as in the detection of changes in symptoms from baseline, which could facilitate improved information for diagnostic assessment, but may also potentially be adapted for use in treatment monitoring. Studies have also shown that EMA technology is more effective at predicting suicidal ideation and behavior than more infrequent, in-person assessments [31-33]. Women also mentioned having someone to reach out and talk to when there is a need as a benefit of the app. Previous studies have shown that an mHealth app could be used as a method for delivering appropriate support at the right time and place, thereby enhancing opportunities for improved personalized medicine [30].

Regarding challenges, several women mentioned their own or their partner’s potential concern about sharing mental health data through an mHealth app. Previous research has described similar concerns [15,34]. Given the sensitivity of the medical information collected via EMA technology, it is important to establish adequate protocols to secure and prevent breaches of protected health information. As reported by other studies, providers in this study mentioned the compliance with and sustainability of EMA technology for a long period as important challenges [11,25]. This warrants discussions on the long-term utility of an app such as this for assessing health. However, it is encouraging that none of our participants mentioned any issues with responding to a maximum of 2 questions a day during their pregnancy and postpartum period. Another important, but less discussed, challenge mentioned by one of the women participants is the compatibility of mobile phone apps, given that cell phone companies continuously update their “ios or android” platform. This will require an expert IT professional within the app development team to update and modify the app as per the phone’s operating system updates and assist women and providers to ensure smooth running of the app.

All the women participants reported smartphone use, and several used different apps to monitor pregnancy, child development, and menstruation, indicating the comfort and popularity of smartphone-based health apps among people. However, unlike previous studies, none of our participants mentioned internet or data plan availability as barriers. This could have been due to the dramatic increase in the number of Americans who own a smartphone, from 35% in 2011 to 85% in 2021 [35]. Furthermore, pregnant and postpartum women may consider their smartphone as an economic, a convenient, and an effective means of staying connected with their providers, friends, and family during a period when they may mostly be housebound taking care of their newborn. This could also explain the lack of mention of any issues related to smartphone or internet access and the associated cost by our participants. However, we acknowledge that this may not be the case with all women; hence, discussions related to the accessibility and affordability of internet should be held with prospective users.

### Limitations

We acknowledge that the study sample for the FGDs only included either pregnant or postpartum women; therefore, not all findings may be generalizable to those with other conditions or those who are not in their pregnancy or postpartum period. In addition, a notable limitation of this study is the smaller sample size, which is characteristic of the qualitative research methodology. Furthermore, all participants (women and providers) were from the same geographic area; hence, the results may not be generalizable without further evaluation using a nationally representative sample. Despite these limitations, the key strength of this study is that it provided an in-depth understanding of the various mobile app features that are acceptable and beneficial from the perspective of stakeholders, namely women who are or were pregnant and providers from a variety of clinical settings.

### Conclusions

Women and providers in this study indicated that it is acceptable to use an mHealth app to monitor mood during pregnancy and the postpartum period. Both groups reported that an mHealth app could promote early symptom recognition and timely intervention for maternal mental health disorders. The participants indicated that customization, assurance regarding data security, and health improvements due to app use were the important determinants of acceptance. The long-term use and sustainability of an mHealth app such as this warrant further discussion and research.

## Abbreviations

EMA: ecological momentary assessment
FG: focus group
FGD: focus group discussion
IRB: institutional review board
LSW: licensed social worker
mHealth: mobile health
OBGYN: obstetrician-gynecologist

## Data Availability

The data sets generated and analyzed during this study are available from the corresponding author upon reasonable request.
